# Large HBV Surface Protein-Induced Unfolded Protein Response Dynamically Regulates p27 Degradation in Hepatocellular Carcinoma Progression

**DOI:** 10.3390/ijms241813825

**Published:** 2023-09-07

**Authors:** Yixiao Guo, Jie Shao, Renyu Zhang, Mingwei Han, Lingmin Kong, Zekun Liu, Hao Li, Ding Wei, Meng Lu, Shuai Zhang, Cong Zhang, Haolin Wei, Zhinan Chen, Huijie Bian

**Affiliations:** National Translational Science Center for Molecular Medicine and Department of Cell Biology, Fourth Military Medical University, Xi’an 710032, China; g2624845095@163.com (Y.G.);

**Keywords:** large HBV surface protein, chronic ER stress, p27, selective translation, ubiquitination, HRD1, hepatocellular carcinoma

## Abstract

Up to 50% of hepatocellular carcinoma (HCC) is caused by hepatitis B virus (HBV) infection, and the surface protein of HBV is essential for the progression of HBV-related HCC. The expression of large HBV surface antigen (LHB) is presented in HBV-associated HCC tissues and is significantly associated with the development of HCC. Gene set enrichment analysis revealed that LHB overexpression regulates the cell cycle process. Excess LHB in HCC cells induced chronic endoplasmic reticulum (ER) stress and was significantly correlated with tumor growth in vivo. Cell cycle analysis showed that cell cycle progression from G1 to S phase was greatly enhanced in vitro. We identified intensive crosstalk between ER stress and cell cycle progression in HCC. As an important regulator of the G1/S checkpoint, p27 was transcriptionally upregulated by transcription factors ATF4 and XBP1s, downstream of the unfolded protein response pathway. Moreover, LHB-induced ER stress promoted internal ribosome-entry-site-mediated selective translation of p27, and E3 ubiquitin ligase HRD1-mediated p27 ubiquitination and degradation. Ultimately, the decrease in p27 protein levels reduced G1/S arrest and promoted the progress of HCC by regulating the cell cycle.

## 1. Introduction

Hepatitis B virus (HBV) infection is a major and strong risk factor for hepatocellular carcinoma (HCC), contributing to approximately 50% of HCC incidence [1]. It has been reported that more than 2 billion people worldwide have been exposed to HBV and nearly 296 million people were chronic HBV carriers by 2022 [2]. HBV is a circular double-stranded DNA virus that consists of four partially overlapping open reading frames (ORFs): pre-S/S, preCore/Core, Pol, and X. The pre-S/S gene contains three inframe start codons that divide the gene into the pre-S1, pre-S2, and S regions, which encode three forms of the hepatitis B surface proteins, large HBV surface protein (LHB), middle HBV surface protein (MHB), and small HBV surface protein (SHB), respectively [3]. All three forms are cotranslationally inserted into the endoplasmic reticulum (ER) as transmembrane proteins and assemble into hepatitis B surface antigen (HBsAg), which envelops the viral nucleocapsid in appropriate proportions [4]. These proteins share a common carboxyl terminus, but their amino termini are different. SHB contains the S region, MHB contains the pre-S2 and S regions, and LHB contains the pre-S1, pre-S2, and S regions. A meta-analysis involving 43 studies and 11,582 patients infected with HBV showed that pre-S mutations were associated with a 3.77-fold increased risk of HCC [5]. HBV pre-S mutations are present in approximately 63% of patients with HCC [6].

Pre-S mutations, deletions, or insertions may lead to the accumulation of surface proteins in the endoplasmic reticulum (ER) and secretory defects, resulting in the failure of these three forms to assemble into virus particles [7]. Gross glassy hepatocytes (GGHs) are characterized by a significant accumulation of viral surface proteins in the ER and are considered a pathological hallmark of chronic HBV infection [8,9]. In previous studies using HBV transgenic mouse models with LHB overproduction, the appearance of GGHs was observed in the liver of mice. When LHB production increases, the serum HBsAg concentration decreases significantly due to the accumulation of unsecreted HBsAg in the ER of hepatocytes, causing the ER to expand to form ground-glass cells [10,11,12]. It was noted that GGHs represent preneoplastic lesions and accelerate HCC development [13] and LHB accumulation in the ER, which is accelerated by pre-S mutation, inducing ER stress that contributes to hepatocarcinogenesis [10,14,15]. ER stress may activate the unfolded protein response (UPR) signaling pathways, which induce genomic instability, oxidative stress, and metabolic switching during tumor progression [16,17].

Transformation of the hepatocytes to HBV-associated HCC is usually accompanied by inflammation, fibrosis, and cirrhosis, and HBV dysregulates the cell cycle during the process of HCC development. Since strong and sustained hepatocellular proliferation preceding hepatocarcinogenesis was observed in LHB-hepatic loading transgenic mouse models, the pathogenesis of LHB-driven UPR signaling in disease progression needs to be fully understood.

In the present study, we demonstrated that LHB is highly expressed in HBV(+) HCC and is closely related to the clinical prognosis of HCC patients. Excess LHB accumulation in the ER causes chronic ER stress, which regulates p27 transcription through activation of the UPR signaling pathway, followed by selective translation of p27 mediated by internal ribosome entry sites (IRESs), and ER-related degradation (ERAD) ultimately enhances p27 ubiquitination, resulting in a significant reduction in p27 protein levels. As a cell cycle inhibitor, the decrease of p27 accelerates HCC progression. Our study proposes a carcinogenic effect of LHB that depends on the precise regulation of cell cycle progression in a chronic ER stress-dependent manner. This study helps to elucidate the mechanisms by which LHB promotes the tumorigenesis of HCC and reveals potential targets for the prevention and treatment of HBV-related HCC.

## 2. Results

### 2.1. LHB Is Upregulated in Human HBV(+) HCC Tissues and Associated with Poor Prognosis

**Figure 1:**
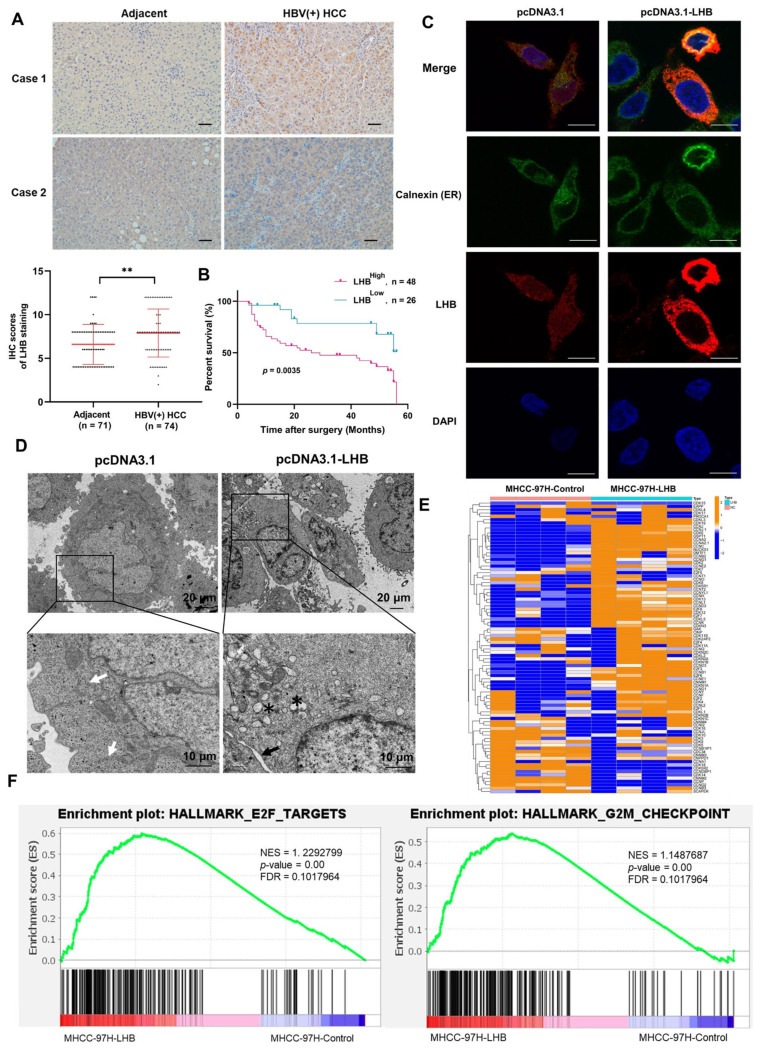
Intracellular retention of LHB leads to morphological changes of ER and affects the cell cycle of HCC. (**A**) The expression level of LHB in tumor and adjacent tissues of HBV(+) HCC patients was analyzed by IHC (*n* = 74) and representative cases are shown. Scale bars: 100 μm. (**B**) Overall survival rates of individuals with high and low LHB expression levels were determined by Kaplan–Meier analysis. (**C**) The localization of LHB in MHCC-97H cells transfected with LHB plasmid was detected by confocal immunofluorescence. Red: LHB; Green: ER. Scale bars: 10 μm. (**D**) Transmission electron microscopy was performed to observe the morphological changes of ER in MHCC-97H cells transfected with LHB plasmid. The white arrows indicate normal ER, the black arrow indicates swollen ER, and the black asterisks represent broken ER. (**E**) Heat map showing the expression of genes related to cell cycle caused by LHB overexpression. (**F**) Gene set enrichment analysis (GSEA) showed that G2/M phase of the cell cycle and E2F transcription factor family were enriched in LHB-overexpressing cells. Two-tailed Student’s *t* test was used to test the significance of differences between two groups; data are represented as mean ± SD. ** *p* < 0.01.

To investigate the expression of LHB in HBV(+) HCC patients and its relationship with HCC prognosis, we used immunohistochemistry to analyze the expression of LHB in 74 pairs of HBV(+) HCC and adjacent tissues. We observed that the expression of LHB in HCC tissues was significantly higher than that in adjacent tissues (Figure 1A). Further analysis of the effect of LHB expression on the clinical prognosis of HCC patients showed that the overall survival of HCC patients with high LHB protein levels was shorter than that of HCC patients with low LHB expression levels (*p* = 0.0035, Figure 1B). The correlation analysis between LHB expression and clinicopathological features showed that the high-LHB phenotype was significantly associated with tumor differentiation (*p* = 0.0054) (Table 1). These results suggest that LHB is related to the development and progression of HCC.

### 2.2. Intracellular Retention of LHB Leads to Morphological Changes of ER and Affects the Cell Cycle of HCC

HBV variants with point mutations, deletions, or insertions in the pre-S sequences are often found in patients with long-lasting chronic hepatitis B. The point mutation involving the start codon of the pre-S2 with consequent complete abolishment of the MHB protein synthesis was constructed [18]. We constructed a plasmid in which the ATG start codons of MHB and SHB were mutated to ACG so that LHB was expressed as the only HBV surface gene, and a specific antibody was designed targeting the pre-S1 region for the detection of LHB, which has a molecular weight of 45 kDa (Figure S1). Subsequently, the specificity of the antibody was confirmed by dot blotting (Figure S2A). To investigate the molecular mechanism by which LHB promotes HCC, we constructed LHB expression plasmids and overexpressed them in two cell lines, HCCLM3 and MHCC-97H. Moreover, we established two stable cell lines, MHCC-97H-LHB and HCCLM3-LHB, through lentivirus infection (Figure S2B). Notably, the MHCC-97H cell line is derived from human HCC cell line MHCC97, which was isolated from a 39-year-old male. HBV DNA is integrated into the cell genome. Thus, endogenous LHB was detected in MHCC-97H controls using our produced LHB antibody [19,20]. Immunofluorescence staining of cells transfected with the vector showed a diffuse, punctate distribution of LHB, whereas LHB aggregated in the cytoplasm as coarse particles and colocalized with the ER marker calnexin in HCC cells overexpressing LHB (Figure 1C). Electron microscopy showed that the ER in LHB-overexpressing cells was swollen and broken, and the proportion of abnormal ER was significantly higher than that in the control group, which is probably the morphological manifestation of an excessive protein load in the ER that disrupts the normal biological function of the ER (Figure 1D and Figure S2C).

Next, we extracted total RNA from MHCC-97H-LHB cells and performed RNA sequencing (RNA-seq). RNA-seq analysis showed a total of 58 genes upregulated and 30 genes downregulated among genes associated with the cell cycle (Figure 1E and Supplementary Table S2). Gene set enrichment analysis (GSEA) showed that LHB over-expression significantly affected the cell cycle of HCC cells, manifested by accelerated G2/M transition and elevated expression of members of the E2F transcription factor family (Figure 1F).

### 2.3. LHB Induces ER Stress and Promotes Tumor Formation by Regulating the Cell Cycle

**Figure 2:**
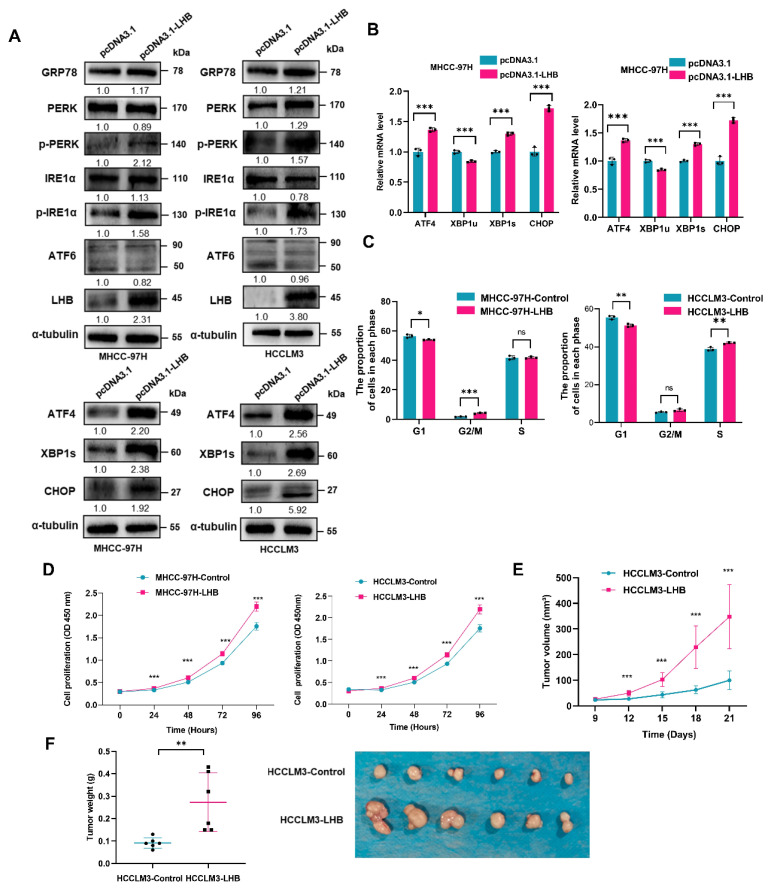
LHB induces ER stress and promotes tumor formation by regulating the cell cycle. (**A**) The expression of UPR-related proteins in HCC cells after overexpression of LHB was determined by Western blotting. (**B**) The mRNA expression of transcription factors ATF4, XBP1s, and CHOP in HCC cells transfected with LHB was detected by qRT-PCR. (**C**) The cell cycle distribution of LHB stably transfected MHCC-97H and HCCLM3 cell lines was analyzed by flow cytometry. (**D**) CCK-8 assay was used to assess the cell proliferation of LHB stably transfected MHCC-97H and HCCLM3 cell lines. (**E**,**F**) HCCLM3-LHB cells stably transfected with LHB were subcutaneously injected into nude mice (*n* = 6). The tumor size of the mice was measured every three days, and the tumor weight was measured on day 21. The experimental results were representative of three independent experiments. Two-tailed Student’s *t* test was used to test the significance of differences between two groups; data are represented as mean ± SD. * *p* < 0.05; ** *p* < 0.01; *** *p* < 0.001; ns, not significant.

The Western blotting analysis showed that the protein expression level of UPR-related genes and the phosphorylation level of IRE1α and PERK increased significantly (Figure 2A). ATF6p50, XBP1s, and ATF4 regulate the transcription of target genes related to ER stress [21]. In this study, the protein and mRNA levels of ATF4, XBP1s, and CHOP, which are downstream of the UPR signaling pathway, increased strikingly in LHB-overexpressing cells (Figure 2A,B). Cell cycle analysis indicated that G1 arrest was reduced and the proportion of cells in the G2 and M phases increased in the MHCC-97H-LHB and HCCLM3-LHB cell lines (Figure 2C and Figure S2D). CCK-8 assays showed that LHB overexpression enhanced cell proliferation (Figure 2D). We conducted cell cycle and cell proliferation experiments to explore the effect of 4-PBA, an ER stress inhibitor, on the growth of HCC cells. The results showed that 4-PBA could arrest the G1 to S phase transition in HCC cells overexpressing LHB (Figure S2E). Furthermore, 4-PBA significantly inhibited the proliferation of HCC cells overexpressing LHB (Figure S2F).

To further explore the role of LHB in vivo, we constructed a xenograft nude mouse model by subcutaneously inoculating HCCLM3-LHB cells into nude mice. Overexpression of LHB promoted tumorigenesis in xenograft mice, and the tumor volume and weight of the LHB overexpression group were significantly higher than those of the control group (Figure 2E,F). The expression of LHB in mouse tumors was evaluated by immunohistochemistry (Figure S2G). These results indicate that LHB accumulated in the ER, causing ER stress and promoting tumor formation by regulating the cell cycle.

### 2.4. Overexpression of LHB Regulates Cell Cycle Progression by Enhancing p27 Ubiquitination

**Figure 3:**
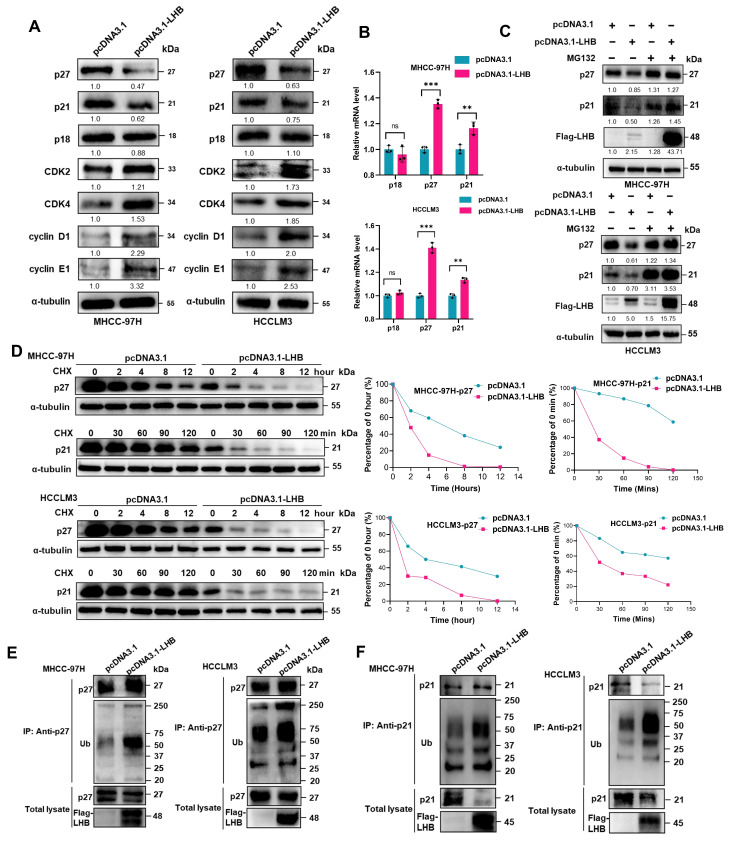
LHB regulates the cell cycle process by enhancing ubiquitination of p27 and p21. (**A**) Western blotting was used to determine the expression of cyclin and cyclin-dependent kinases (CDK), and CDK inhibitors. (**B**) The mRNA expression of cell cycle inhibitors was analyzed by qRT-PCR. (**C**) Western blotting detected the expression of p27 and p21 in LHB-overexpressing HCC cell lines after treatment with MG132 (10 μM) for 12 h. (**D**) HCC cell lines transfected with LHB plasmid were treated with cyclohexylamine (CHX) (100 μg/mL), and protein lysates were collected at specified time for analysis of half-lives by Western blotting. (**E**,**F**) The ubiquitination of p27 and p21 in HCC cell lines with LHB over-expression was determined by immunoprecipitation and Western blotting. The experimental results were representative of three independent experiments. Two-tailed Student’s *t* test was used to test the significance of differences between two groups; data are represented as mean ± SD. ** *p* < 0.01; *** *p* < 0.001; ns, not significant.

Next, we explored the molecular mechanism by which LHB accelerates the cell cycle, especially G0/G1 phase transition. We examined the protein expression of cyclins and cyclin-dependent kinases, including CDK2, CDK4, cyclin D1, and cyclin E1, which are particularly important for the transition from G1 to S phase. In addition, the cyclin-dependent kinase inhibitors p18, p27, and p21 inhibit cell cycle progression to S phase. As shown in Figure 3A,B, the expression of cyclins and CDKs, positive growth regulators, increased significantly. Interestingly, we found that p27 and p21 protein expression was dramatically reduced, but their mRNA expression was significantly increased in LHB-overexpressing cells. In contrast, the expression of p18 was not affected by LHB overexpression. We investigated whether the differences in the mRNA and protein expression of the CDK inhibitors p21 and p27 were caused by specific degradation. We found that the degradation of the p21 and p27 proteins in HCC cells overexpressing LHB was inhibited by treating with MG132, a proteasome inhibitor (Figure 3C). To determine whether LHB overexpression increases the instability of the p21 and p27 proteins, we treated LHB-overexpressing MHCC-97H and HCCLM3 cell lines with cycloheximide (CHX) to monitor the rate of protein degradation. Western blotting analysis showed that the degradation rate of p27 and p21 after LHB overexpression was accelerated, and the half-life of these proteins became shorter (Figure 3D). This conclusion was further confirmed by the enhanced ubiquitination of p27 and p21 in MHCC-97H and HCCLM3 cell lines overexpressing LHB (Figure 3E,F and Figure S3). Our results showed that LHB increased the expression of p27 and p21 mRNA and then enhanced the ubiquitination and subsequent degradation of p27 and p21, significantly decreasing their protein levels. The instability of p21 and p27 results in an increase in the G1/S phase transition during the cell cycle.

### 2.5. LHB-Induced ER Stress Regulates p27 mRNA Transcription and Enhances the Selective Translation of p27

**Figure 4:**
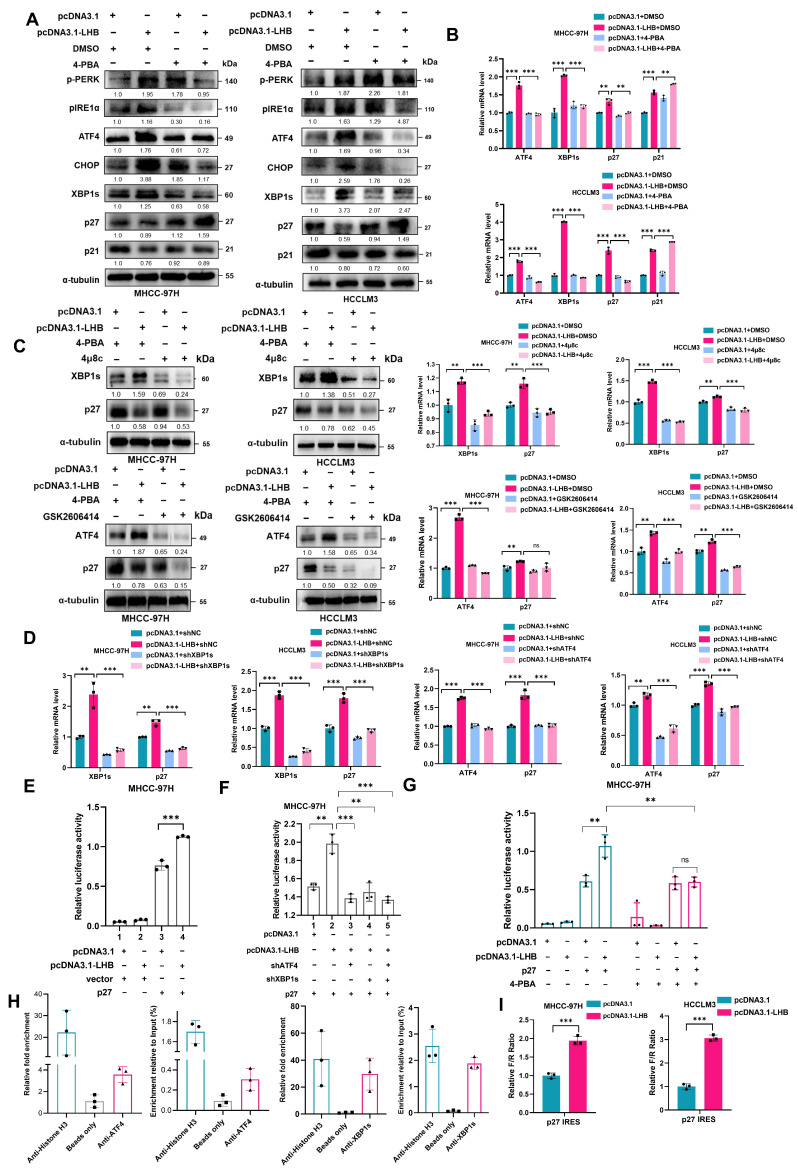
LHB-induced ER stress regulates the transcription of p27 and the selective translation of p27. (**A**) Western blotting was used to analyze the protein expression of p27, p21, and ER stress-related markers in HCC cell lines with the overexpression of LHB treated with 4-PBA (1 mM) for 24 h. (**B**) The relative mRNA levels of ER stress-related transcription factors, p27, and p21 in LHB-overexpressed cell lines treated with 4-PBA (1 mM) for 24 h were determined using qRT-PCR. (**C**) Western blotting and qRT-PCR detected the expression of p27 in LHB-overexpressing HCC cell lines with the treatment of 4μ8c (30 μM) or GSK2606414 (20 μM) for 24 h. (**D**) The mRNA expression of p27 was detected by qRT-PCR in HCC cell lines co-transfected with LHB and shATF4 or shXBP1s. (**E**) Dual-luciferase reporter assay detected the promoter activity of p27 induced by LHB in MHCC-97H cells. (**F**) Dual-luciferase reporter assay detected the promoter activity of p27 in MHCC-97H cells co-transfected with human p27 promoter (−2000/+100) plasmid, LHB plasmid, and shATF4 or shXBP1 plasmids. (**G**) MHCC-97H cells with LHB overexpression were treated with 4-PBA (1 mM) for 24 h, and p27 promoter activity was detected by dual-luciferase reporter assay. (**H**) ChIP assay detected ATF4 and XBP1s binding to the promoter of p27 in MHCC-97H cells. Relative fold enrichment was calculated based on qRT-PCR results compared with the control beads. (**I**) pR-p27-F and LHB plasmids were co-transfected into HCC cell lines, and the ratio of firefly luciferase activity to Renilla was measured. The experimental results were representative of three independent experiments. Two-tailed Student’s t test was used to test the significance of differences between two groups; data are represented as mean ± SD. ** *p* < 0.01; *** *p* < 0.001; ns, not significant.

To further explore the crosstalk between ER stress induced by LHB and the cell cycle, HCC cell lines overexpressing LHB were treated with 4-PBA, an ER stress inhibitor. Western blotting analysis showed that ER stress was inhibited and the decrease in p27 protein expression was reversed, but the expression of the p21 protein did not change obviously (Figure 4A). Meanwhile, under the condition of ER stress inhibition, the expression of p27 mRNA decreased significantly, whereas the expression of p21 mRNA still increased markedly (Figure 4B). These data indicate that LHB-induced ER stress promoted progression of the cell cycle by regulating the expression of p27 but not p21. As shown in Figure 2A, LHB-induced ER stress activated the PERK and IRE1α signaling pathways; we next asked whether p27 was transcriptionally regulated by the transcription factors downstream of these two signaling pathways. After treatment with 4μ8c, which is an inhibitor of the IRE1α pathway, we found that the expression level of p27 mRNA decreased in HCC cell lines overexpressing LHB (Figure 4C). The mRNA expression level of p27 was also decreased after treatment with the PERK pathway inhibitor GSK2606414 (Figure 4C). Western blotting analysis showed that the expression of p27 decreased more obviously after treatment with 4μ8c and GSK2606414 (Figure 4C and Figure S4A).

We further verified that p27 was regulated by ATF4 and XBP1s, which are the primary transcription factors of downstream of the PERK and IRE1α pathways. First, we found the overexpression of ATF4 and XBP1s in HCC cell lines increased the mRNA and protein levels of p27 significantly (Figure S4B). More interestingly, LHB overexpression dramatically increased the mRNA expression of p27, which was suppressed after silencing ATF4 or XBP1s (Figure 4D and Figure S4C). Collectively, these results indicate that p27 was regulated by both ATF4 and XBP1s. To support this conclusion, a dual-luciferase reporter assay was performed in MHCC-97H cells, and the results showed that ATF4 and XBP1s significantly increased the promoter activity of p27 (Figure S5A). Then, we verified the transcriptional activity of p27 in MHCC-97H cells overexpressing LHB. As expected, the transcriptional activity of p27 increased significantly with LHB overexpression (Figure 4E). After silencing of ATF4 and XBP1s, LHB-stimulated p27 transcriptional activity decreased significantly (Figure 4F). To further study the effect of ER stress on p27 transcriptional activity, we treated LHB-overexpressing MHCC-97H cells with the ER stress inhibitor 4-PBA, and the results showed that the transcriptional activity of p27 markedly decreased (Figure 4G). Thus, we concluded that the ER stress induced by LHB increased the transcriptional activity of p27.

To evaluate the binding of ATF4 and XBP1s to the p27 promoter region in HCC cells, we predicted binding sites using the JASPER website and then verified them by ChIP analysis. We found that ATF4 could bind to the promoter regions of p27 genes in MHCC-97H cells and that this binding was enhanced by ATF4 overexpression. The transcription factor XBP1s also bound to the promoter region of p27 (Figure 4H and Figure S5B). Previous studies have shown that under ER stress, the translation of most proteins is suppressed, except for a few proteins that can be translated through IRESs to maintain essential biological activities [22]. To evaluate whether p27 translation can be regulated by LHB-induced ER stress, we constructed and validated a dual-luciferase reporter plasmid for detecting the IRES activity of the p27-5’UTR (Figure S5C). We observed that LHB overexpression significantly increased the activity of the p27 IRES-controlled luciferase reporter (Figure 4I). It is reported that eIF3 not only plays an important role in cap-dependent translation, it also interacts with RNA stem-loop in the 5’UTR of mRNAs to directly recruit ribosomes [23]. We therefore explored whether eIF3d, the major RNA binding subunit of the eIF3 complex, is involved in IRES-dependent translation of p27 under chronic ER stress induced by LHB. The results showed that knockdown of eIF3d significantly reduced LucR activity, but did not affect LucF activity, which indicated that the level of cap-dependent translation is reduced, but IRES-mediated p27 translation level is not changed (Figure S6A,B). These data show that ER stress induced by LHB promoted the selective translation of p27. Collectively, these results implied that LHB induced ER stress and activated the UPR signaling pathway to regulate p27 transcription; subsequently, p27 was selectively translated in an IRES-dependent manner to increase its expression level.

### 2.6. p27 Degradation by the E3 Ubiquitin Ligase HRD1 Occurs in the Presence of LHB-Induced ER Stress

**Figure 5:**
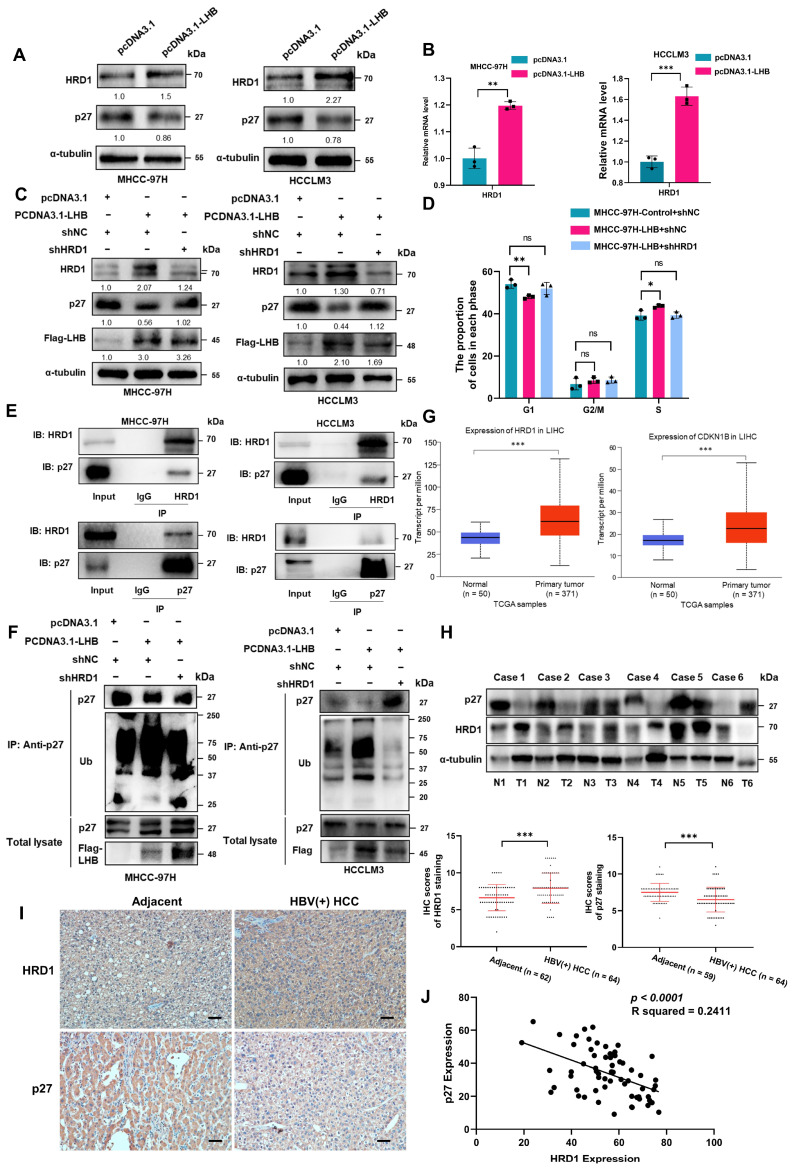
p27 is degraded by the E3 ubiquitin ligase HRD1. (**A**) The expression of HRD1 in HCC cell lines overexpressing LHB was detected using Western blotting. (**B**) Expression of HRD1 in HCC cell lines overexpressing LHB was detected by qRT-PCR. (**C**) The expression of p27 in HCC cell lines co-transfected with LHB and shHRD1 plasmids was analyzed by Western blotting. (**D**) Cell cycle distribution in MHCC-97H-LHB cells silenced with shHRD1 was analyzed by flow cytometry. (**E**) Endogenous interaction between HRD1 and p27 in HCC cell lines was detected by coimmunoprecipitation and Western blotting. (**F**) Immunoprecipitation and Western blotting were performed to detect ubiquitination of p27 in HCC cell lines co-transfected with LHB and shHRD1 plasmids. (**G**) The mRNA expression levels of HRD1 and p27 in liver cancer were analyzed based on TCGA database. (**H**) Western blotting was used to detect p27 and HRD1 protein expression in six pairs of human HCC and matched adjacent tissues. T, HCC tissues; N: adjacent tissues. (**I**) The expression levels of HRD1 and p27 in tumor and adjacent tissues of HBV(+) HCC patients were analyzed by IHC and representative cases were shown (*n* = 64). Scale bar: 50 μm. (**J**) Pearson’s correlation test was used to evaluate the correlation between p27 expression and HRD1 expression in in 64 patients with HBV(+) HCC. The experimental results were representative of three independent experiments. Two-tailed Student’s *t* test was used to test the significance of differences between two groups; data are represented as mean ± SD. * *p* < 0.05; ** *p* < 0.01; *** *p* < 0.001; ns, not significant.

ER stress usually leads to activation of the ERAD pathway. ERAD is a protein quality control system that evolved to maintain ER homeostasis in cells. HRD1 is an E3 ubiquitin ligase that spans the ER membrane. HRD1 forms a complex with its stabilizing factor SEL1L, representing the most evolutionarily conserved ERAD machinery, whose best-characterized function is to catalyze the degradation of misfolded/unfolded proteins and proteins that degrade quickly [24,25]. We speculated that the degradation of p27 might involve ERAD. Next, we investigated whether the p27 protein level is regulated by the ERAD pathway. In fact, when LHB was overexpressed, the protein and mRNA expression levels of the ERAD-associated HRD1 were significantly increased (Figure 5A,B). Further study of the role of HRD1 in accelerating the progression of HCC after overexpression of LHB showed that the protein expression of p27 increased significantly with HRD1 silencing (Figure 5C). Cell cycle analysis showed that in MHCC-97H-LHB cells, G1/S arrest increased after HRD1 silencing (Figure 5D and Figure S7). Coimmunoprecipitation assays showed that HRD1 could interact with p27 in HCC cells and HEK293T cells (Figure 5E and Figure S8A). Moreover, the ubiquitination of p27 was obviously enhanced when LHB was overexpressed, which was reduced by silencing HRD1 (Figure 5F). Furthermore, we analyzed the mRNA expression of HRD1 and p27 in HCC and adjacent nontumor tissues using a TCGA data set; the number of HCC tissue samples and adjacent nontumor samples was 371 and 50, respectively. The results showed that the mRNA expression of HRD1 and p27 in HCC was increased compared with that in adjacent tissues (Figure 5G). In the tumor samples from HCC patients with positive HBV, there was no difference in p27 mRNA expression between HCC and adjacent tissues, but the protein expression level was significantly decreased in HCC tissues (Figure 5H and Figure S8B). In contrast, the mRNA and protein expression of HRD1 increased significantly (Figure 5H and Figure S8B). Next, we analyzed the expression of HRD1 and p27 in 64 pairs of HBV(+) HCC and adjacent tissues by IHC and observed that the expression of HRD1 in HCC was higher than that in adjacent tissues. Consistently, p27 expression was significantly decreased in HCC compared with paired adjacent tissues (Figure 5I). IHC showed that the expression levels of HRD1 and p27 were negatively correlated in HBV(+) HCC patients (Figure 5J). Collectively, these results indicate that HRD1 is an E3 ubiquitin ligase of p27 that accelerates the ubiquitination and degradation of p27, thus regulating the cell cycle in HCC cells and promoting tumor growth.

### 2.7. p27 Expression Is Regulated by LHB-Induced ER Stress In Vivo

**Figure 6:**
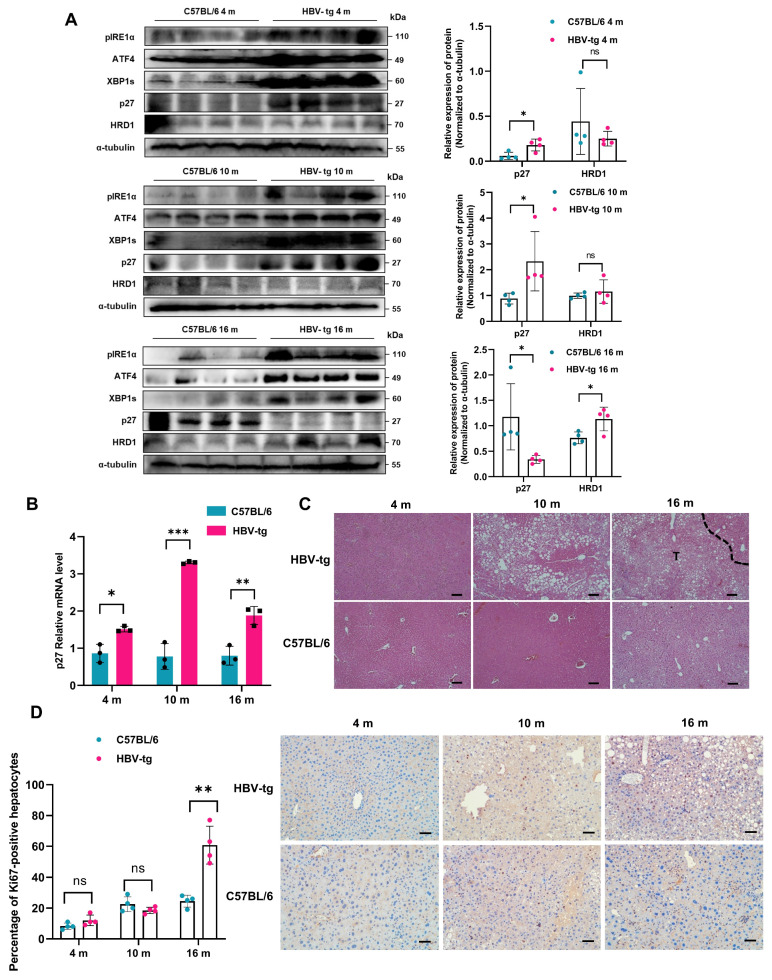
LHB-induced chronic ER stress regulates the expression of p27 in HBV-tg mice. (**A**) The expression of ER stress-related transcription factors, p27, and HRD1 in liver tissues from 4-, 10-, and 16-month-old HBV-tg mice was analyzed by Western blotting. (**B**) The relative mRNA levels of p27 in HBV-tg transgenic mice at different stages was determined using qRT-PCR. (**C**) H&E staining was used to analyze the damage of liver tissue from HBV-tg mice at 4, 10, and 16 months old. Scale bars: 50 μm. (**D**) The percentage of Ki67 in hepatocytes from HBV-tg mice and age control mice was analyzed by IHC. The percentage of nuclear Ki67-positive hepatocytes of each group (*n* = 4) was quantified and representative cases are shown. Scale bars: 50 μm. The experimental results were representative of three independent experiments. Two-tailed Student’s *t* test was used to test the significance of differences between two groups; data are represented as mean ± SD. * *p* < 0.05; ** *p* < 0.01; *** *p* < 0.001; ns, not significant.

We further verified the molecular mechanism by which LHB promotes the progression of HCC in vivo. An HBV-tg mouse model with overexpression of the LHB gene in hepatocytes was used (Figure S9). After 16 months of feeding, a spontaneous model of hepatitis-liver fibrosis-HCC progression was established. According to the progression of HCC, we divided the transgenic mice into three groups: a 4-month-old hepatitis group, a 10-month-old liver fibrosis group, and a 16-month-old HCC group. As shown in Figure 6A, the protein expression of the transcription factors ATF4 and XBP1s increased in the liver tissues from three groups of HBV-tg mice compared with the age control mice, indicating sustained and chronic ER stress. However, the protein expression of p27 increased in 4- and 10-month-old HBV-tg mice, and markedly decreased at 16 months compared with age control mice. More importantly, the protein expression of HRD1 increased significantly only in 16-month-old HBV-tg mice, whereas it showed no differences in 4- and 10-month-old HBV-tg mice compared with age control mice. The expression of p27 mRNA in the liver tissues from HBV-tg mice aged 4 months, 10 months, and 16 months increased significantly compared with age control mice (Figure 6B). At the same time, H&E staining showed more severe liver tissue damage in HBV-tg mice than in their littermates (Figure 6C). As shown in Figure 6D, IHC analysis showed that the expression of Ki67 in hepatocytes was significantly higher in 16-month-old HBV-tg mice than in age control mice, but was comparable in paired 4-month-old mice and paired 10-month-old mice, indicating that the enhanced cell proliferation had occurred at 16 months, a stage of tumorigenesis, and implying that the HRD1-p27 axis contributes to HCC progression after 10 months.

**Figure 7:**
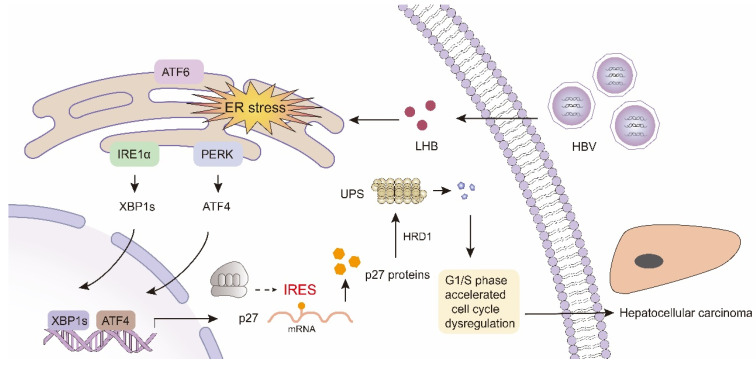
A model of LHB promoting HCC tumorigenesis by inducing chronic ER stress to regulate consecutive events of p27 mRNA expression, IRES-mediated translation, and ubiquitination degradation.

Our data thus far suggested that p27 undergoes transcriptional regulation, selective translation, and ubiquitination degradation by HRD1 in HBV-infected HCC patients, and this process is ER stress-dependent. Collectively, our data demonstrated that the accumulation of LHB in the ER induced ER stress, which triggered the activation of the PERK and IRE1α pathways, and their downstream transcription factors ATF4 and XBP1s upregulated the mRNA of p27. Subsequently, p27 was selectively translated by mediation of IRESs. Ultimately, the E3 ligase HRD1 enhanced the ubiquitination of p27, which resulted in a decrease in p27 expression and accelerated cell cycle progression in HCC (Figure 7).

## 3. Discussion

In the present study, we revealed a pattern of sequential transcription-selective translation–ubiquitination of the tumor suppressor gene p27 that promotes HCC tumorigenesis via LHB-induced chronic ER stress. Several novel findings came out of this work. First, we propose that LHB, as a cancer-promoting factor, induces chronic ER stress by directly activating UPR signaling during cell proliferation by accelerating the cell cycle G1/S phase transition. Second, we verified that ATF4 and XBP1s, transcription factors of downstream of the PERK and IRE1α pathways, directly regulate p27 transcription. Third, we showed that the transcribed p27 mRNA could be selectively translated under ER stress, which was mediated by the IRES sequence located in the p27-5′UTR. Fourth, the translated p27 protein was ubiquitinated and subsequently degraded by the ERAD-related E3 ligase HRD1. Thus, our study provides a novel mechanism by which HBV surface proteins promote HCC tumorigenesis through the precise regulation of CDK inhibitors under chronic pathological ER stress.

Previous studies have shown that, compared with wild-type mice, HBsAg transgenic mice are more likely to develop HCC, which may due to the high expression of the oncogenes Bim1 and Dkk1. HBV particles derived from cell culture can promote the proliferation and colony formation of hepatoma cell lines [26]. A recent study showed that in HBsAg transgenic mice, the accumulation of HBsAg induces inactivation of Hippo signaling and upregulation of BMI1, and increased BMI1 directly mediates cell proliferation related to the decrease in levels of p16, p19 and p53 [27]. Moreover, HBV X protein is related to the cell cycle progression and hepatocarcinogenesis of HCC, and the mitochondrial protein SIRT4 has been demonstrated to upregulate the expression of p16 and p21 proteins, suppress cyclin B1/Cdc2 and Cdc25c, and suppress survivin to induce cell apoptosis [28]. HBV surface proteins are indispensable for the biological activity of HBV and have been identified as potential targets for antiviral therapy. These surface proteins have been reported to be associated with ER stress signaling pathways [29]. HBV surface proteins are translated by cotranslocation in hepatocytes, and appropriate folding and posttranslational modifications, such as glycosylation, are carried out in the ER. LHB is a form of HBV surface protein that is closely related to ER stress in HCC. Previous studies have demonstrated that in patients with persistent HBV infection, many mutations, including deletions and mutations, are present in the pre-S1 and pre-S2 regions [30]. LHB with pre-S deletions or mutations results in defective secretion of viral particles and induction of ER stress due to SHB accumulation in the ER, and histopathological changes are observed as GGHs [17]. Current studies on the mechanisms by which LHB with pre-S mutations accelerates the progression of the cell cycle in HCC include investigations of direct binding to JAB1 (Jun activation domain binding protein 1), which leads to degradation of the oncogene p27, cyclin A upregulation, and induction of hepatocyte nodal proliferation through an ER stress-independent mechanism [31,32]. Nevertheless, how LHB directly utilizes the UPR signaling pathways to regulate p27 expression has not been elucidated. In this study, we found that LHB was associated with poor prognosis in HCC. Interestingly, further studies revealed that excess LHB aggregates in the ER and the resulting induced chronic ER stress promotes the cell cycle in HCC cells through the precise regulation of p27, thus accelerating hepatocarcinogen activity.

ER stress triggers the activation of the UPR signaling pathway, a network of pathways aimed at reestablishing homeostasis [33]. Indeed, cell fate determination appears to depend on the intensity and duration of the UPR, with acute ER stress and chronic ER stress playing opposite roles due to cellular adaptation. Previous studies have shown that chronic ER stress is hepatotoxic and contributes to the development of chronic liver diseases [17,34]. Consistent with previous reports, our data in this study showed that LHB-induced ER stress promoted the proliferation of HCC cells. We suggest the existence of LHB-induced chronic ER stress, in which the response switches from initial adaptive survival to signaling favoring tumor survival. We confirmed that this adaptive signaling conversion process is dependent on ERAD. E3 ligases play an important organizational role in ERAD, and more than a dozen ERAD E3 ligases have been identified thus far [35]. In our study, HRD1 was found to play an important role as an E3 ligase of p27 in the regulation of p27 protein levels during the development of HCC in HBV-tg mice.

Paired transcriptional and proteomic data from 159 patients were examined, yielding 6203 mRNA–protein pairs in which the levels of genes involved in the cell cycle are weakly correlated, suggesting that cell cycle-related molecules are primarily post-transcriptionally regulated [36]. Studies have shown that p27 levels are largely attributed to posttranslational modifications, such as phosphorylation and ubiquitination, ultimately leading to proteasomal degradation [37]. We found that overexpression of LHB enhanced the ubiquitination and degradation of p27 in HCC cells. In this study, we used LHB transgenic mice capable of spontaneous HCC formation and investigated the activation of the UPR signaling pathway and the expression of p27 at different stages of the HCC development process. We found a dynamic process of p27 protein expression. This interesting process involves the upregulation of p27 transcription, IRES-dependent selective translation of p27 under stress, and degradation of p27 by the ERAD-related E3 ligase HRD1. One result of p27 instability is increased G1/S phase transition and acceleration of the cell cycle. ER stress, ERAD, and IRES constitute a mechanism for fine regulation of p27. Our research enriches our understanding of the molecular mechanism by which chronic ER stress regulates the cell cycle phase and promotes the progression of liver cancer. Studies have shown that ER stress is closely associated with the development of many diseases, such as neurodegenerative and metabolic diseases [38,39], and other important studies on ER stress have focused on solid tumors. In this context, we suggest that chronic ER stress and ERAD are possible targets for the treatment of HBV(+) HCC, and the regulation of molecules other than p27 that are closely related to HCC progression needs to be further explored. We also found that p18, p21, and p27 together act as cell cycle inhibitors at the G1 phase of the cell cycle and that excessive LHB accumulation in HCC cells promoted the ubiquitination of p21 and p27 but had no significant effect on p18. A poorly characterized role of p18 in cell cycle progression in vivo has been reported [40].

The regulation of p21 may differ from that of p27. One study in oligodendrocytes suggested that p27 is required for proper cell cycle exit, whereas p21 is not [41]. The existence of IRES elements in cell mRNA was investigated by high-throughput strategy, and it was shown that 10% of mRNAs could potentially be translated by a cap-independent mechanism [42]; over 100 proposed IRES-containing mRNAs have been reported. At present, an online website (http://bioinfo.net.in/IRESPred, accessed on 21 October 2022) can predict whether a gene 5’UTR has the structure of IRES. The p21 5’UTR was predicted to contain IRES elements while the p18 5’UTR did not contain IRES elements. This finding suggested that LHB-induced ER stress activated the UPR signaling pathway, and its transcription factors ATF4 and XBP1s regulated mRNA expression of p27 and p21, both of which were translated due to the IRES element in the mRNA 5’UTR of p27 and p21. The results in Figure 3C demonstrated that the degradation of both p27 and p21 protein was regulated by the ubiquitination–proteasome system; however, only the p27 degradation was closely related to LHB-induced ER stress (Figure 4A). It is reported that LHB activates GSK-3β/ACSL3 signal pathway [43]. Therefore, the ubiquitination-mediated degradation mechanism of p21 by LHB regulation needs to be further explored.

All of the three HBV surface proteins, LHB, MHB, and SHB, are indispensable components of the viral envelope. The retention of the three proteins can trigger ER stress leading to aberrant cell proliferation and angiogenesis in HCC progression [18,44], however, in our study in vivo tumorigenesis in the nude mice model was studied by solely expressed LHB. Thus, further studies are warranted to extensively investigate and compare the tumorigenic potential of the three forms of HBV surface proteins.

## 4. Materials and Methods

### 4.1. Cell Culture and Transfection

The MHCC-97H cell line was obtained from the Cell Bank of the Chinese Academy of Sciences (Shanghai, China). The HCCLM3 cell line was obtained from the BeNa Culture Collection (Suzhou, China). The HEK293T cell line were obtained from the American Type Culture Collection (ATCC, Manassas, VA, USA). Cells were cultured in RPMI 1640 (10–040-CVRC, Corning, Corning, NY, USA) medium supplemented with 10% fetal bovine serum (10100-147, Gibco, Waltham, MA, USA), 2% L-glutamine, 1% penicillin, and streptomycin in an incubator with 5% carbon dioxide, at 37 °C and 95% humidity. Plasmids, siRNAs, or shRNAs were transfected into cells using Lipofectamine 2000 (11668019, Invitrogen, Carlsbad, CA, USA) according to the manufacturer’s instructions. Specifically, the cells were transfected at approximately 70% density in the culture plates, and the supernatant was replaced with serum-containing medium 6 h later. The mRNA and protein expression levels of the transfected cells were assessed at 24 or 48 h after transient transfection. Sequences of the siRNAs and shRNAs used in this study are listed in Supplementary Materials Table S1.

### 4.2. Immunohistochemistry

Immunohistochemistry (IHC) experiments were used to detect the expression of LHB, p27, and HRD1 in the tissue microarray of HCC (Shanghai Outdo Biotech Co., Shanghai, China). Paraffin sections were dewaxed first, and antigen retrieval was performed with citrate buffer solution at pH 6.0. The sections were sealed and incubated with the primary antibody in a moist chamber at 4 °C overnight. After incubation with primary antibodies, the sections were stained with a streptavidin peroxidase staining kit (Zhongshan Jinqiao Co., Beijing, China). The nuclei were stained with hematoxylin, and the tissues were sealed after alcohol dehydration. All IHC was independently assessed by two experienced pathologists or analyzed for integrated optical density by using Image-Pro Plus Version 6.0 software (Media Cybernetics Inc., Bethesda, MD, USA). All images were measured by using the same conditions [45]. The parameters used for evaluation were the staining intensity and the area of positive staining, and the final scores were then calculated. The staining intensities were graded as 0 (no staining), 1 (weak), 2 (moderate), and 3 (strong). The scores for the positive staining area were as follows: 0 (0–25%), 1 (26–50%), 2 (51–75%), and 3 (76–100%).

### 4.3. Transgenic Mice Model

HBV-transgenic [C57BL/6J-Tg (Alb1HBV)44Bri/J] mice (HBV-tg) were purchased from the Jackson Laboratory, and the sales agency was Beijing Vital River Laboratory Animal Technology Co., Ltd., Beijing, China (002226). By placing the sequence encoding large HBV surface protein downstream of the mouse albumin promoter, mice were induced to overexpress large HBV surface protein in hepatocytes. Tails of two-week old mice were cut, and genomic DNA was extracted. DNA was subsequently amplified by conventional PCR and the amplified products were detected by agarose gel electrophoresis.

### 4.4. In Vivo Xenograft Mouse Mode

The ethical approval for the animal experiment was obtained from the ethics committee and institutional review committee of the National Center for Translational Science of Molecular Medicine of Fourth Military Medical University (2022-NTSCMM-ID006). Six-week-old male nude mice were obtained from Beijing Vital River Laboratory Animal Technology Co., Ltd., Beijing, China. After feeding for one week, mice were subcutaneously injected with 5 × 10^6^ HCCLM3-LHB cells or HCCLM3-Control cells. The tumor size was measured every three days from the ninth day. Three weeks later, the mice were sacrificed by cervical dislocation, the subcutaneous tumors were dissected, the size and weight of the tumors were measured, and the tumors were photographed. The tumors were soaked in 10% formalin and sectioned for IHC.

### 4.5. Plasmid Construction

The online website (https://www.ncbi.nlm.nih.gov/nuccore/MN172185.1, accessed on 10 January 2021) was used to obtain the sequence of the HBV pre-S/S gene. The mutation sequence of the HBV pre-S/S gene is listed in Supplementary Figure S1. HBV-LHB covered the entire coding region of the pre-S1, pre-S2, and S genes. The initiating two ATG codons of the MHB and SHB were both mutated to ACG when constructing the LHB plasmids; therefore, the plasmid expressed only LHB but not MHB or SHB. LHB was constructed by inserting the PCR-generated LHB fusion gene into Ubi-MCS-3FLAG-SV40-puromycin. The primer sequences for LHB were as follows: Forward: 5′-CCA ACT TTG TGC CAA CCG GTC GCC ACC ATG GGA GGT TGG TCA TCA AAA C-3′; Reverse: 5′-AAT GCC AAC TCT GAG CTT AAT GTA TAC CCA GAG ACA AAA G-3′. The 5’UTR sequence of p27 was synthesized by GenCript (Nanjing, China). The pR-p27-F plasmid was obtained by inserting the p27 5’UTR between Renilla luciferase (RL) and firefly luciferase (FL) in the dual-luciferase reporter gene carrier pRF. To validate the pR-p27-F plasmid, the pR-Rev p27-F plasmid was constructed by inserting the p27 5’UTR backward between RL and FL as a negative control. To exclude FL expression due to ribosomal read-through, the phR-p27-F recombinant plasmid vector was constructed by adding a segment of DNA to the pRL-p27-F vector.

### 4.6. Reagents

Chemical compounds including 4μ8c (s7272), GSK2606414 (s7307), MG-132 (s2619), and 4-phenylbutyric acid (s3592) were obtained from Selleck.cn (Houston, TX, USA); cycloheximide (CHX, HY-12320) was purchased from MCE company (Newark, NJ, USA).

### 4.7. Real-Time qPCR

Total RNA was extracted with the Total RNA Kit II (Omega, Riverside, CA, USA), and reverse transcription PCR was performed using the PrimeScript RT reagent kit (TaKaRaBio, Otsu, Japan). Subsequently, cDNA was amplified by quantitative RT-PCR using a TB Green PCR kit (TaKaRa, Otsu, Japan). Relative quantities of mRNAs were determined using the comparative threshold number (ΔΔCt method). β-actin was used for qRT-PCR internal reference. The sequences of the primers are listed in Supplementary Table S1.

### 4.8. Western Blotting

Tissues and cells were treated with RIPA for extraction of proteins. Equal amounts of protein sample were added to each well of a 12% SDS-PAGE gel. The protein samples were transferred to PVDF membranes after electrophoresis, blocked with 5% skim milk for 1 h, and then incubated overnight at 4 °C with the corresponding primary antibodies. The next day, the secondary antibodies were incubated at room temperature for another 1 h. The protein signals were visualized using a Western-light chemiluminescence detection system (Image Station 4000 MM Pro, Boston, MA, USA). The antibodies used in this study were as follows: anti-GRP78 (11587-1-AP, 1:1000), an-ti-ATF6 (24169-1-AP, 1:1000), anti-p27 (25614-1-AP, 1:1000), anti-p21 (10355-1-AP, 1:1000), anti-Flag (66008-3-Ig, 1:1000), anti-HRD1 (66045-1-AP, 1:1000), anti-CDK2 (10122-1-AP, 1:1000), anti-CDK4 (11026-1-AP, 1:1000), anti-cyclin D1 (26939-1-AP, 1:1000), anti-cyclin E1 (11554-1-AP, 1:1000), and anti-alpha Tubulin (66031-1-Ig, 1:1000) purchased from Proteintech (Wuhan, China); anti-ATF4 (11815s, 1:1000), anti-XBP1s (27901s, 1:1000), anti-CHOP (2895s, 1:1000), anti-Ki67 (12202, 1:1000), and anti-ubiquitin (3936s, 1:1000) obtained from CST (Boston, MA, USA); and anti-PERK (sc-13073, 1:1000), anti-IRE1 (sc-390960, 1:1000), and anti-LHB (sc-57761, 1:1000) purchased from Santa Cruz Biotechnology (Dallas, TX, USA). Anti-LHB antibody was also produced and obtained from ABclonal (Wuhan, China); anti-phospho-IRE1α (ab124945, 1:1000) and anti-p18 (ab192239, 1:1000) were obtained from Abcam (Cambridge, UK). Anti-phospho-PERK (PA5-40294, 1:1000) was obtained from Invitrogen (Carlsbad, CA, USA).

### 4.9. RNA-Seq

RNA-Seq was performed by Gene Denovo (Guangzhou, PR China). MHCC-97H-Control and MHCC-97H-LHB overexpressing LHB cells were lysed using Trizol, and samples were sent to Gene Denovo. Gene set enrichment analysis (GSEA) (version 3.0, MIT and Harvard University, http://software.broadinstitute.org/gsea/downloads.jsp, assessed on 1 May 2022) was performed to explore the biological functions in the development of HCC affected by LHB overexpression.

### 4.10. Cell Proliferation and Cell Cycle Assays

Cells were seeded in 96-well plates at 3000 cells per well, and then 10 μL CCK-8 reagent (Catalog No. C0005, TargetMoI, Boston, MA, USA) was added to each well. After incubation for 2 h in the incubator, the absorbance values were recorded at 450 nm using an EnSpire^®^ (Austin, TX, USA) multimode plate reader. The transfected HCC cells were collected after treatment with specific drugs for 24 h and then fixed with 70% cold ethanol at 4 °C for 6 h. After washing twice with PBS, the cells were incubated with 50 µL RNase A (KGA521, Keygen, Jiangsu, China) at 37 °C for half an hour, then resuspended with 200 µL propidium iodide (PI), and stained in the dark for half an hour. Cells were analyzed with BD FACS Calibur™ flow cytometry (BD Bioscience; Franklin Lake, NJ, USA). The relationship between the PI fluorescence signal of the fl2-a peak and the count was used to determine the cell cycle distribution.

### 4.11. Immunofluorescence

For immunofluorescence in this study, the cells were seeded and transfected with the indicated plasmids in a 35 mm confocal dish for 48 h. Then, the cells were fixed with 4% formaldehyde for 15 min at room temperature and permeabilized with 0.2% Triton X-100 for 8 min. After blocking with 5% goat serum for 50 min, the antibodies were added and incubated overnight at 4 °C. The next day, the cells were incubated with the corresponding fluorescent antibodies at room temperature for 1 h. The nuclei were stained with 4,6-diamino-2-styryl alcohol (DAPI, Beyotime) for 20 min. Images were obtained by using a confocal microscope (Nikon, Melville, NY, USA).

### 4.12. Luciferase Reporter Assay

The transcriptional activity of ATF4 and XBP1s was detected by a p27-luciferase reporter assay. The p27-luciferase reporter (pGV238-p27) was purchased from Genechem Co., Ltd. (Shanghai, China). In 24-well plates, cells were cultured to approximately 70% confluence and then cotransfected with p27-luciferase reporter and Renilla luciferase reporter plasmid (pRL-CMV, Promega, Madison, WI, USA). Twenty-four hours after transfection, luciferase activity was measured with the Luciferase Reporter Assay System (Promega) according to the manufacturer’s instructions.

### 4.13. Chromatin Immunoprecipitation (ChIP)

Cell cycle analyses of MHCC-97H and A549 cells were performed by flow cytometry. Briefly, cells were pretreated with 70% ethanol overnight at 4 °C. After being centrifuged and washed with PBS three times, cells were incubated with cell cycle analysis kit (Keygen, KGA512) reagents for 30 min at room temperature. The distribution of cell cycle phases and percentages were analyzed by FACS Calibur flow cytometer (BD Biosciences, San Jose, CA, USA).

### 4.14. Immunoprecipitation and Coimmunoprecipitation

According to the manufacturer’s instructions, a coimmunoprecipitation kit (26149, Thermo Fisher Scientific, Waltham, MA, USA) was used to detect the interaction between HRD1 and p27 in MHCC-97H, HCCLM3, and HEK293T cells, and an immunoprecipitation kit (26147, Thermo Fisher Scientific, USA) was used to detect the ubiquitination of p27 and p21. After the antibodies were coupled with the resin, the cell lysate was added and shaken overnight in a refrigerator at 4 °C. The protein on the resin was eluted with elution buffer, the effluent was collected the next day, and the results were analyzed by Western blotting. The p21 and p27 antibodies were crosslinked to protein A/G resin for IP. MHCC-97H and HCCLM3 cells were treated with IP lysis buffer, and the cell lysate was added to a centrifugal column containing resin, and incubated at 4 °C for 1 h, while the column was gently turned over to mix well. The flowing liquid was retained as the pretreated cell lysate, which was then added to the centrifugal column, gently turning at 4 °C and mixing overnight. The flowing liquid was collected for detection of the ubiquitination of p21 and p27 in vitro.

### 4.15. Statistical Analysis

All results of statistical analyses are expressed as the mean ± SD. Data from at least three independent experiments are displayed in dot graphs. Data were analyzed using GraphPad Prism 8.0 software (GraphPad Software, La Jolla, CA, USA). The overall survival rate was calculated using Kaplan–Meier analysis. The clinical features of HCC patients and their relationships with LHB protein expression were evaluated by χ^2^ tests. Pearson’s correlation test was used to evaluate the correlation between p27 expression and HRD1 expression in HBV(+) HCC patients. *p* values were determined by two-tailed Student’s t test. *p* < 0.05 was considered statistically significant.

## 5. Conclusions

Overall, our study shows that LHB induces chronic ER stress and that the activated UPR signaling pathway promotes HCC progression. Upon activation of the two UPR signaling pathways PERK and IRE1α, p27 is transcriptionally upregulated by ATF4 and XBP1s, and the IRES-mediated translation of p27, which is eventually degraded by HRD1, the E3 ligase of ERAD, leads to a dynamic decrease in p27 expression. The role of chronic ER stress in this regulatory process is crucial and is thus expected to be a new direction and approach for the treatment of HCC via interference with ER stress and thus tumor progression.

## Figures and Tables

**Table 1:** Clinical features and LHB protein expression in patients with HCC (*n* = 74).

Clinicopathological	LHB Protein Express		*p* Value
Value	High (*n* = 48)	Low (*n* = 26)	
Gender			
Male	43	24	0.7022
Female	5	2	
Age			
<50 years	24	11	0.5269
≥50 years	24	15	
Serum AFP			
<20 (ng/mL)	16	11	0.4439
≥20 (ng/mL)	32	15	
Tumor number			
Single	37	24	0.1337
Multiple	10	2	
Tumor size			
<5 cm	29	20	0.1518
≥5 cm	19	6	
TNM stage			
I	28	20	0.1098
II–IV	20	6	
Tumor differentiation			
I–II	17	18	0.0054
III–IV	31	8	

χ^2^ tests were performed for statistical analysis. AFP, α-fetoprotein; TNM, tumor-node metastasis. *p* < 0.05 was considered statistically significant.

## Data Availability

The datasets generated during the current study are available from the corresponding author upon reasonable request.

## Supplementary Materials

The supporting information can be downloaded at: https://www.mdpi.com/article/10.3390/ijms241813825/s1.
