# Supplementary material for: Large HBV Surface Protein-Induced Unfolded Protein Response Dynamically Regulates p27 Degradation in Hepatocellular Carcinoma Progression
Source: Int J Mol Sci. 2023 Sep 7;24(18):13825. doi: 10.3390/ijms241813825 (PMC10530851; doi:10.3390/ijms241813825)
Supplement: Supplementary file 1 [file ijms-24-13825-s001.zip › Supplemental figure legend.pdf]

Supplementary material

Figure S1. LHB expressing plasmid is constructed by mutating ATG codons of MHB and SHB to ACG in HBV pre-S/S gene.

Figure S2. LHB induces ER stress and promotes tumor formation by regulating cell cycle. (A) Dot blotting detected the produced antibody specific to LHB. (B) Two stable HCC cell lines, MHCC-97H-LHB and HCCLM3-LHB, were established through lentivirus infection. (C) Transmission electron microscope was performed to observe the morphological changes of ER in MHCC-97H cells transfected with LHB plasmid. Quantification of proportion of abnormal ER was presented. (D) Cell cycle distribution of HCC cells overexpressing LHB was analyzed by flow cytometry. (E) Cell cycle distribution of LHB overexpressing HCC cell lines treated with 4-PBA (1 mM) was analyzed by flow cytometry. (F) Cell proliferation of LHB overexpressing HCC cell lines treated with 4-PBA (1 mM) was detected by CCK-8. (G)LHB expression in transplanted tumor tissues of nude mice was analyzed by immunohistochemistry. The experimental results were representatives of three independent experiments. Two-tailed Student's t test was used to test the significance of differences between two groups; data are represented as mean ± SD. *$p < 0.05$; **$p < 0.01$; ***$p < 0.001$.

Figure S3. LHB enhances the ubiquitination of p21 and p27 through ubiquitin-proteasome system. (A) Ubiquitination of p21 and (B) p27 in MHCC-97H and HCCLM3 cells after LHB overexpression and treatment with MG132 (10 μM) for 12 hours were detected by western blotting.

Figure S4. LHB regulates cell cycle process by enhancing ubiquitination of p27. (A) Determination of the optimal inhibitory concentrations of 4μ8c and GSK2606414 on UPR signaling in MHCC-97H cells were detected by western blotting. (B) Western blotting and qRT-PCR detected the protein and mRNA expression of p27 after the overexpression of ATF4 and XBP1s in HCC cell lines. (C) Western blotting detected the knockdown of ATF4 or XBP1s in MHCC-97H cells by shATF4 or shXBP1s plasmids. The experimental results were representatives of three independent experiments. Two-tailed Student's t test was used to test the significance of differences between two groups; data are represented as mean ± SD. ***$p < 0.001$.

Figure S5. Transcription factors ATF4 and XBP1s up-regulate p27 transcription. (A) Dual luciferase reporter assay detected the transcriptional activity of p27 with ATF4 or XBP1s overexpression in MHCC-97H cells. (B) ATF4 and XBP1s binding to the promoter of p27 in MHCC-97H cells was detected by ChIP analysis. (C) The pRL-p27-FL, pRL-Rev p27-FL, and phRL-p27-FL plasmids

were constructed and validated in HCC cell lines. Firefly luciferase and renilla luciferase activity was assayed. The experimental results were representatives of three independent experiments. Two-tailed Student's t test was used to test the significance of differences between two groups; data are represented as mean ± SD. $^{**}p < 0.01$; $^{***}p < 0.001$; ns, not significant.

Figure S6. The eIF3d is not involved in IRES-mediated p27 translation. (A) Knockdown of eIF3d was detected by qRT-PCR. (B) Cap-dependent and IRES-mediated translation of p27 after eIF3d knockdown was determined by dual luciferase reporter assay. The experimental results were representatives of three independent experiments. Two-tailed Student's t test was used to test the significance of differences between two groups; data are represented as mean ± SD. $^{*}p < 0.05$; $^{**}p < 0.01$; $^{***}p < 0.001$; ns, not significant.

Figure S7. Flow cytometry detects cell cycle distribution in HCC cells co-expressing LHB and shHRD1 plasmids.

Figure S8. p27 is degraded by the E3 ubiquitin ligase HRD1. (A) Co-immunoprecipitation and western blotting were used to analyze the interaction between p27 and HRD1 in HEK293T cells. (B) The mRNA expression of p27 and HRD1 in 12 pairs of human HCC and matched adjacent tissues was analyzed by qRT-PCR. The experimental results were representatives of three independent experiments. Two-tailed Student's t tests were used to test the significance of differences between two groups; data are represented as mean ± SD.

Figure S9. The genotype of HBV-tg mice is identified. LHB bands with a size of 223 bp were analyzed by agarose gel electrophoresis.
