# Supplementary material for: Large HBV Surface Protein-Induced Unfolded Protein Response Dynamically Regulates p27 Degradation in Hepatocellular Carcinoma Progression
Source: Int J Mol Sci. 2023 Sep 7;24(18):13825. doi: 10.3390/ijms241813825 (PMC10530851; doi:10.3390/ijms241813825)

Figure S1

atgggaggttggtcatcaaaacctcgcaaaggcatggggacgaatctttctgttcccaaccctctgggattctttcccgatc
atcagttggaccctgcattcggagccaactcaaacaatccagattgggacttcaaccccatcaaggaccactggccagca
gccaaccaggtaggagtgggagcattcgggccagggctcacccctccacacggcggtattttggggtggagccctcag
gctcagggcatattgaccacagtgtcaacaattcctcctcctgcctccaccaatcggcagtcaggaaggcagcctactccc
atctctccacctctaagagacagtcatcctcaggcc**acg**cagtggaattccactgccttccaccaagctctgcaggatccca
gagtcaggggtctgtatcttcctgctggtggctccagttcaggaacagtaaaccctgctccgaatattgcctctcacatctcg
tcaatctccgcgaggactggggaccctgtgacgaac**acg**gagaacatcacatcaggattcctaggacccctgctcgtgtt
acaggcggggtttttcttgttgacaagaatcctcacaataccgcagagtctagactcgtggtggacttctctcaattttctag
ggggatctcccgtgtgtcttggccaaaattcgcagtccccaacctccaatcactcaccaacctcctgtcctccaatttgtcctg
gttatcgctggatgtgtctgcggcgttttatcatattcctcttcatcctgctgctatgcctcatcttcttattggttcttctggattat
caaggtatgttgcccgtttgtcctctaattccaggatcaacaacaaccagtacgggaccatgcaaaacctgcacgactcctg
ctcaaggcaactctatgtttccctcatgttgctgtacaaaacctacggatggaaattgcacctgtattcccatcccatcgtcct
gggctttcgcaaaatacctatgggagtgggcctcagtccgtttctcttggctcagtttactagtgccatttgttcagtggttcg
tagggctttcccccactgtttggctttcagctatatggatgatgtggtattgggggccaagtctgtacagcatcgtgagtccc
tttataccgctgttaccaattttcttttgtctctgggtatacatttaa

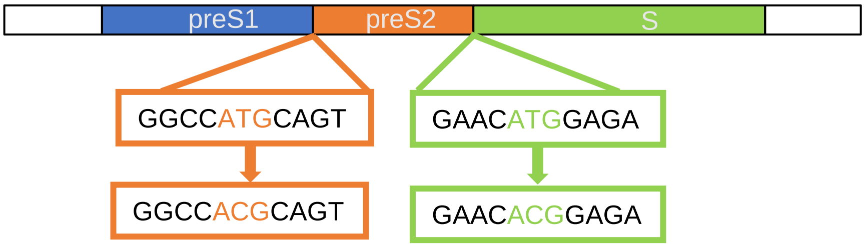

Figure S2

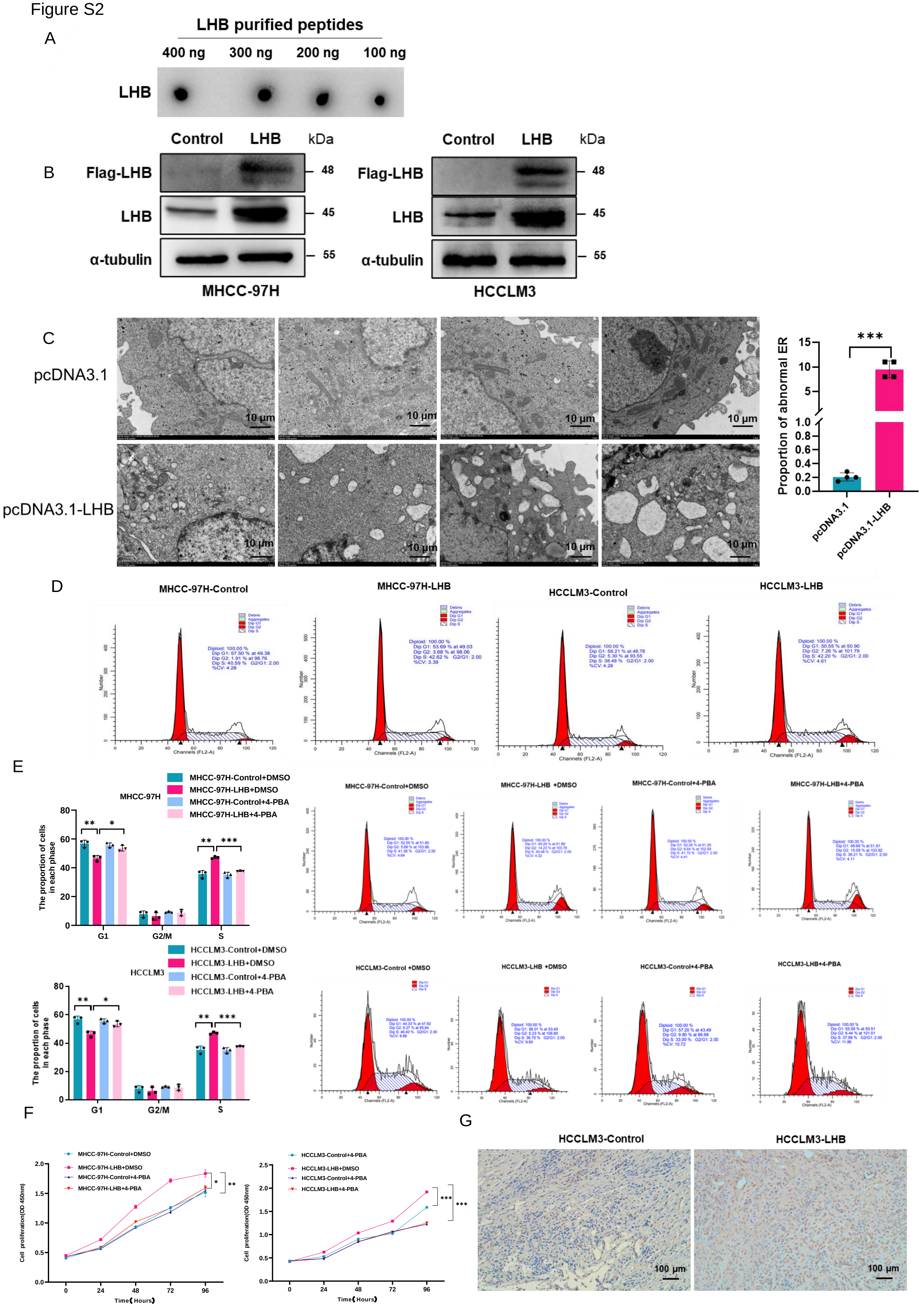
A
LHB purified peptides
400 ng
300 ng
200 ng
100 ng
LHB
B
Control
LHB
kDa
Flag-LHB
LHB
α-tubulin
48
45
55
MHCC-97H
HCCLM3
C
pcDNA3.1
pcDNA3.1-LHB
10 μm
Proportion of abnormal ER
***
D
MHCC-97H-Control
MHCC-97H-LHB
HCCLM3-Control
HCCLM3-LHB
E
MHCC-97H
MHCC-97H-Control+DMSO
MHCC-97H-LHB+DMSO
MHCC-97H-Control+4-PBA
MHCC-97H-LHB+4-PBA
The proportion of cells in each phase
G1
G2/M
S
HCCLM3
HCCLM3-Control+DMSO
HCCLM3-LHB+DMSO
HCCLM3-Control+4-PBA
HCCLM3-LHB+4-PBA
F
Cell proliferation(OD 450nm)
Time(Hours)
G
HCCLM3-Control
HCCLM3-LHB
100 μm

Figure S3

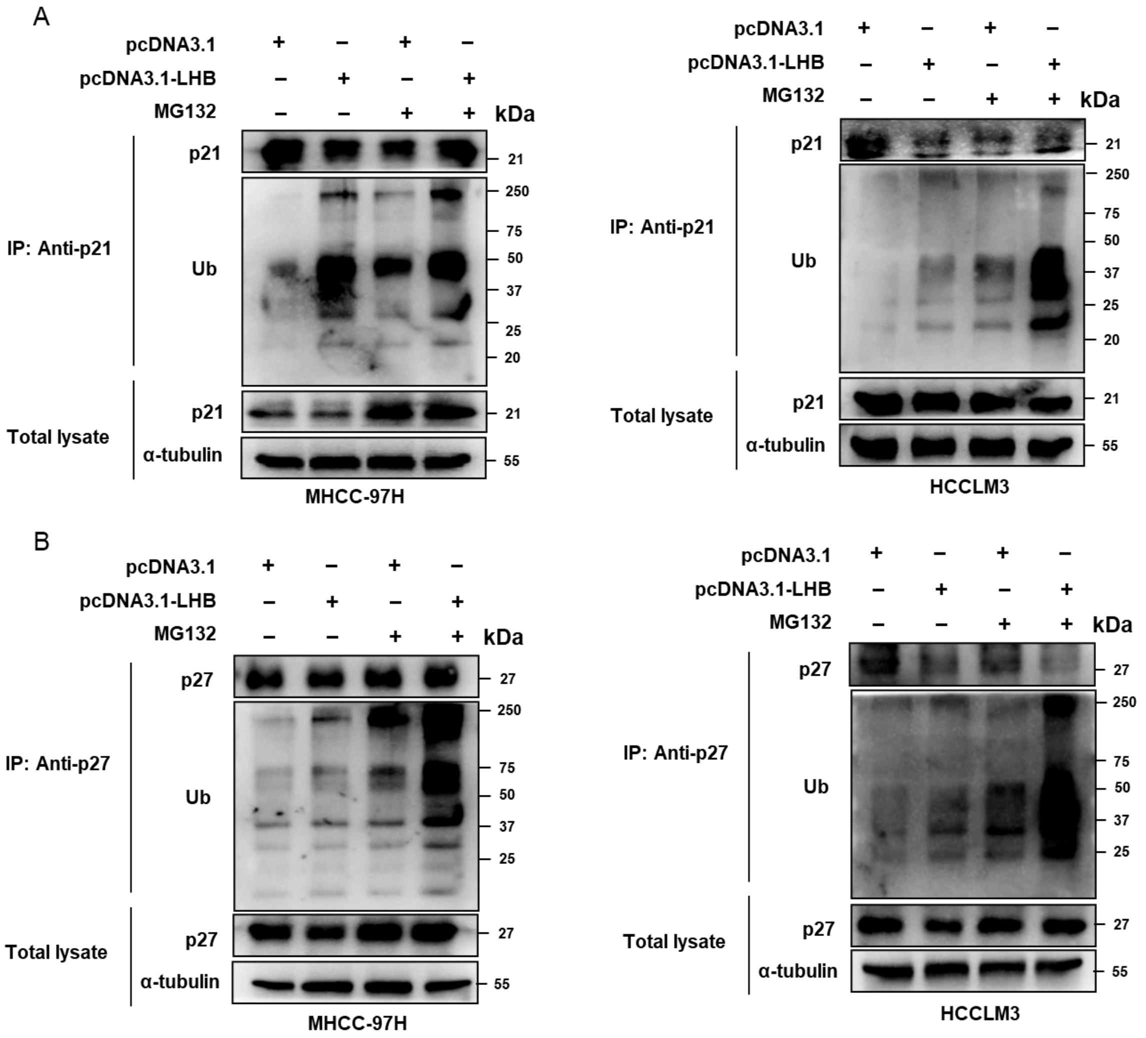
A
pcDNA3.1
pcDNA3.1-LHB
MG132
kDa
IP: Anti-p21
p21
Ub
Total lysate
α-tubulin
21
250
75
50
37
25
20
55
MHCC-97H
HCCLM3
B
IP: Anti-p27
p27
27
MHCC-97H
HCCLM3

Figure S4

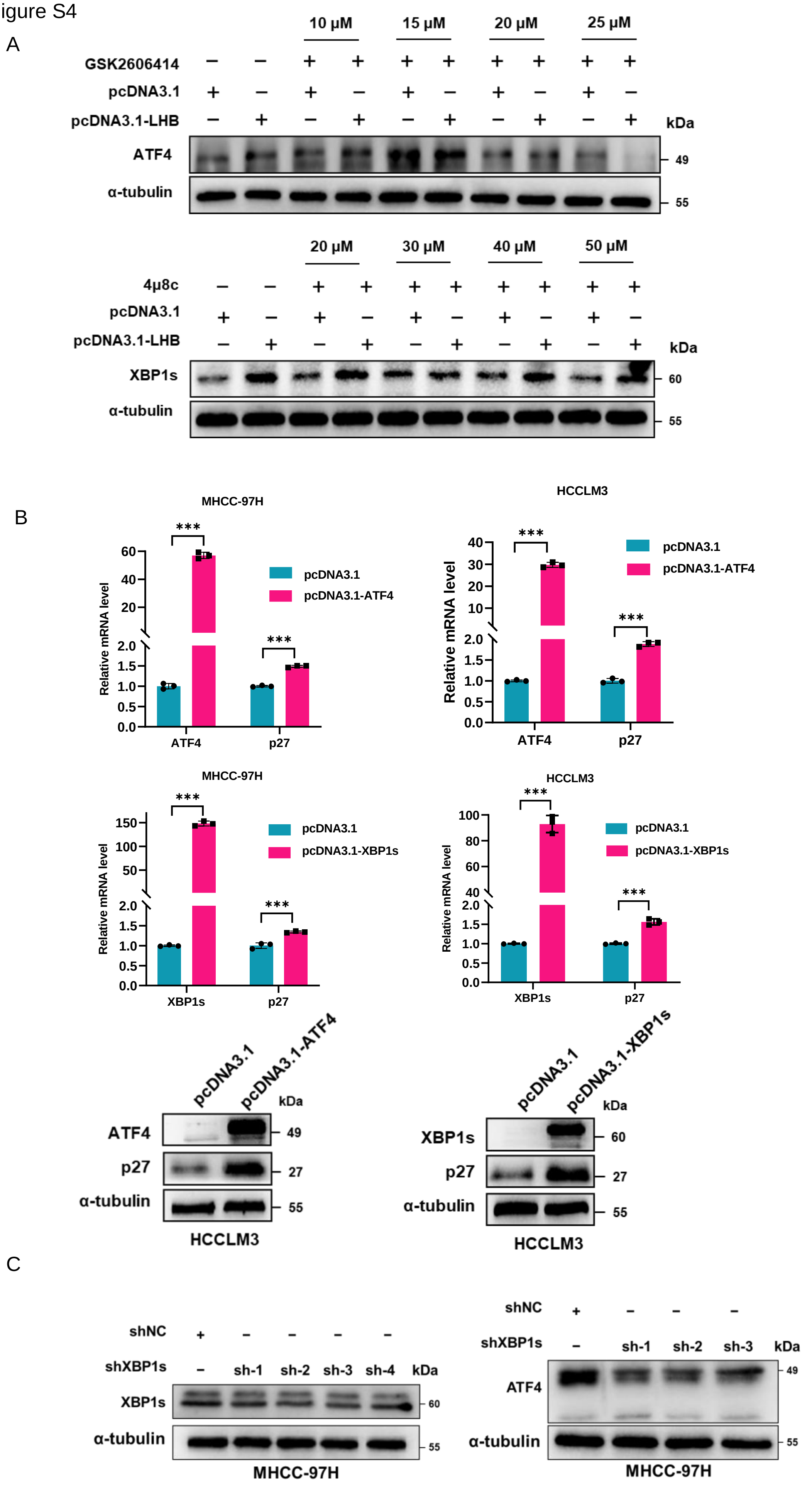
A
10 μM
15 μM
20 μM
25 μM
GSK2606414
pcDNA3.1
pcDNA3.1-LHB
kDa
ATF4
α-tubulin
49
55
20 μM
30 μM
40 μM
50 μM
4μ8c
pcDNA3.1
pcDNA3.1-LHB
kDa
XBP1s
α-tubulin
60
55
B
MHCC-97H
HCCLM3
Relative mRNA level
pcDNA3.1
pcDNA3.1-ATF4
ATF4
p27
pcDNA3.1-XBP1s
XBP1s
pcDNA3.1
pcDNA3.1-ATF4
pcDNA3.1-XBP1s
kDa
ATF4
p27
α-tubulin
XBP1s
49
27
55
60
HCCLM3
C
shNC
shXBP1s
sh-1
sh-2
sh-3
sh-4
kDa
XBP1s
α-tubulin
60
55
MHCC-97H
ATF4
49

Figure S5

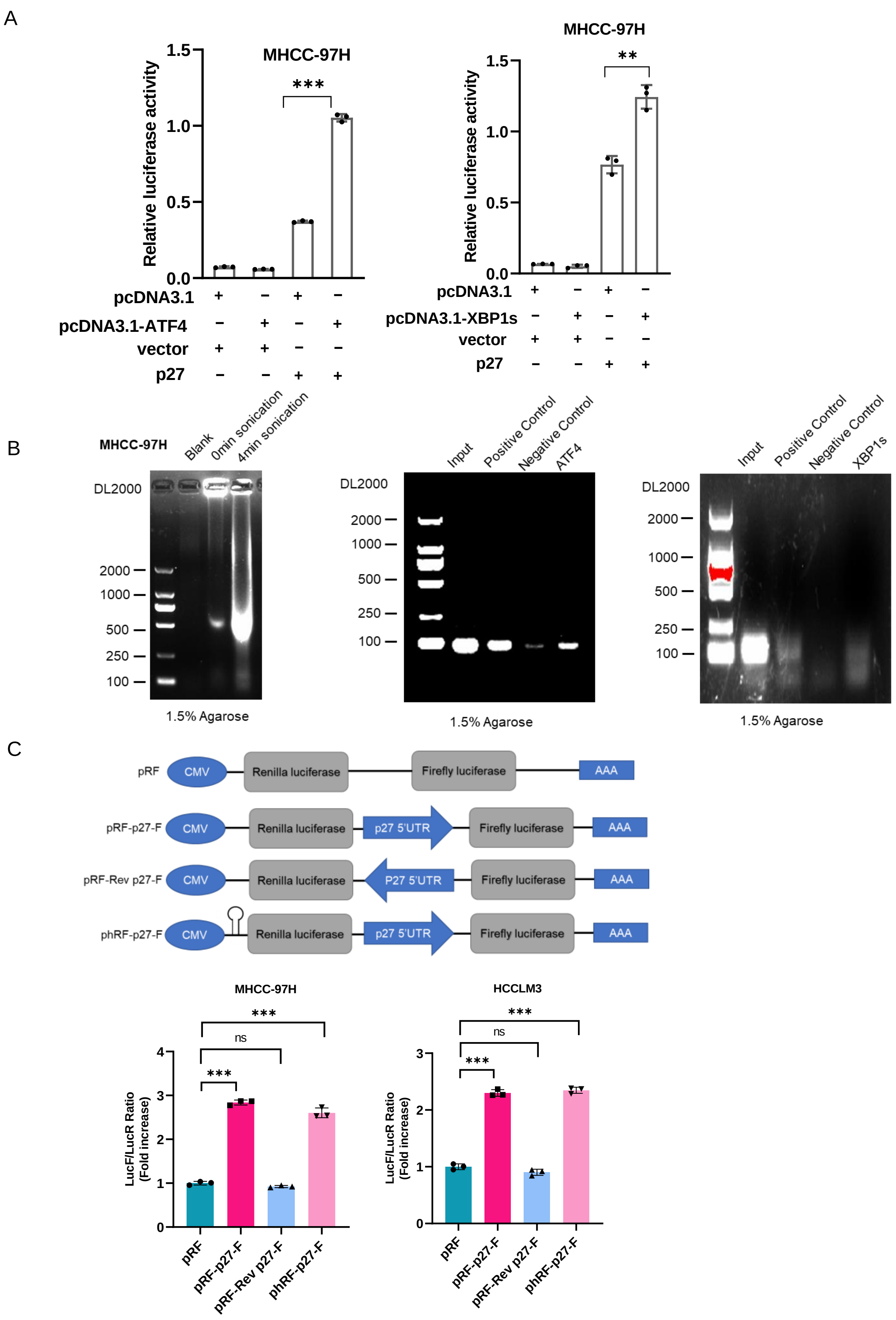

A
MHCC-97H
Relative luciferase activity
***
**
pcDNA3.1
pcDNA3.1-ATF4
pcDNA3.1-XBP1s
vector
p27
B
MHCC-97H
Blank
0min sonication
4min sonication
Input
Positive Control
Negative Control
ATF4
XBP1s
DL2000
2000
1000
500
250
100
1.5% Agarose
C
pRF
pRF-p27-F
pRF-Rev p27-F
phRF-p27-F
CMV
Renilla luciferase
Firefly luciferase
p27 5'UTR
P27 5'UTR
AAA
MHCC-97H
HCCLM3
LucF/LucR Ratio
(Fold increase)
ns

Figure S6

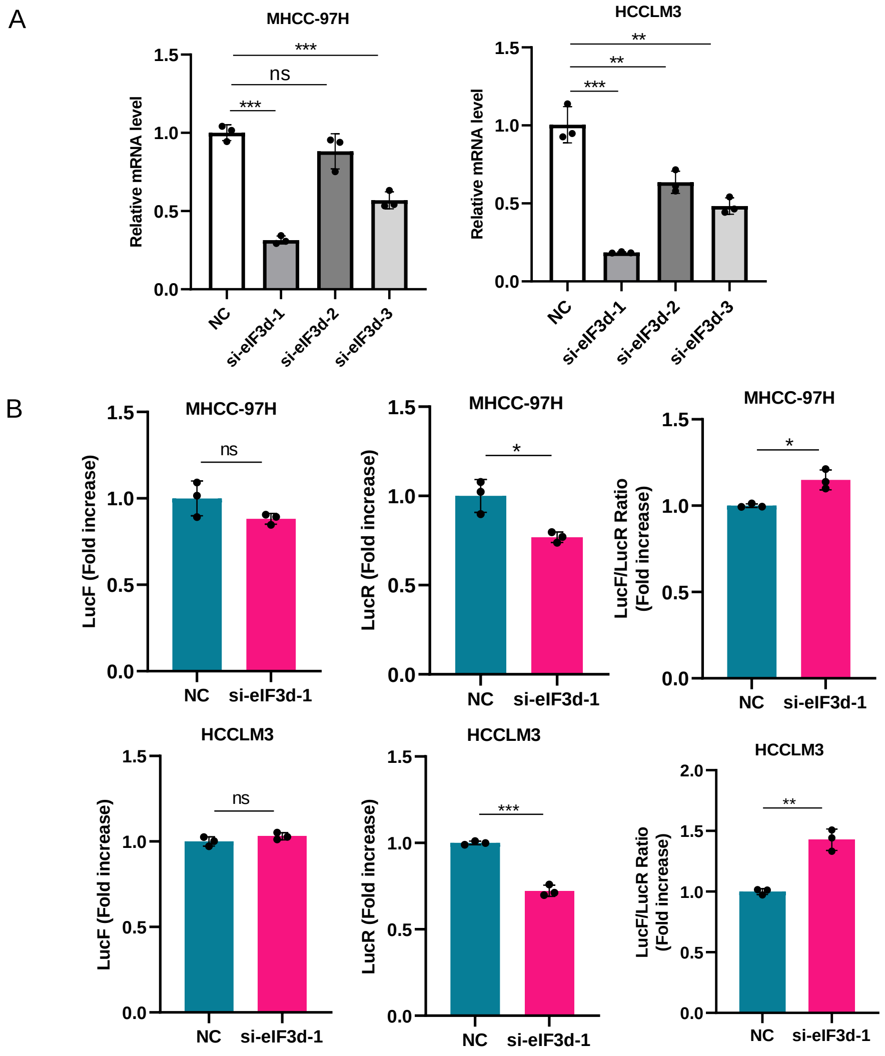
A
MHCC-97H
Relative mRNA level
1.5
1.0
0.5
0.0
***
ns
***
NC
si-eIF3d-1
si-eIF3d-2
si-eIF3d-3
HCCLM3
Relative mRNA level
**
**
***
B
MHCC-97H
LucF (Fold increase)
ns
NC
si-eIF3d-1
LucR (Fold increase)
*
LucF/LucR Ratio
(Fold increase)
HCCLM3
***
**
2.0

Figure S7

MHCC-97H-Control+shNC

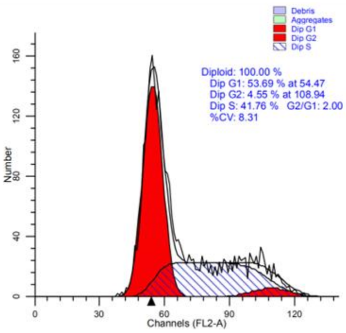

MHCC-97H-LHB+shNC

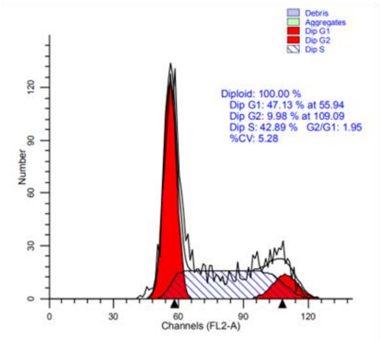

MHCC-97H-LHB+shHRD1

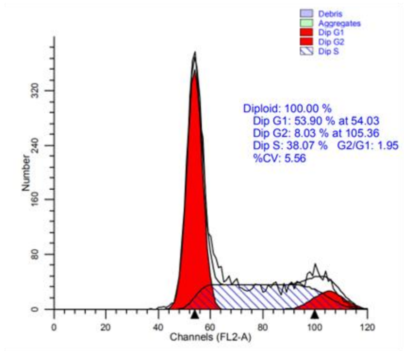

Figure S8

A

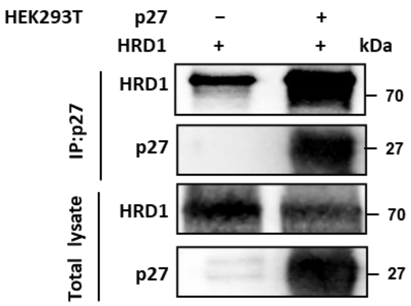

HEK293T
p27 – +
HRD1 + +
kDa
IP:p27
HRD1
70
p27
27
Total lysate
HRD1
70
p27
27

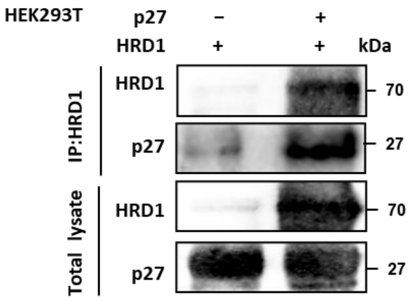

HEK293T
p27 – +
HRD1 + +
kDa
IP:HRD1
HRD1
70
p27
27
Total lysate
HRD1
70
p27
27

B

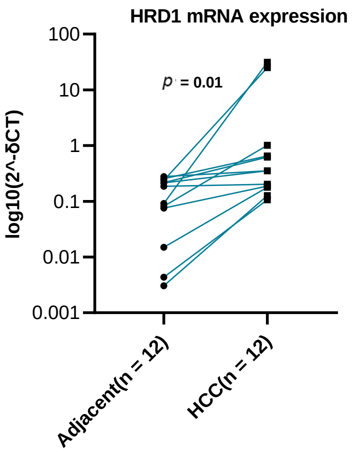

HRD1 mRNA expression
p = 0.01
log10(2^-δCT)
100
10
1
0.1
0.01
0.001
Adjacent(n = 12)
HCC(n = 12)

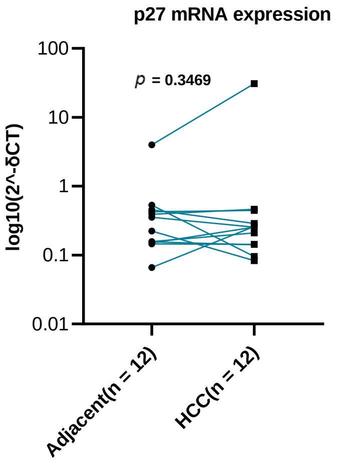

p27 mRNA expression
p = 0.3469
log10(2^-δCT)
100
10
1
0.1
0.01
Adjacent(n = 12)
HCC(n = 12)

Figure S9

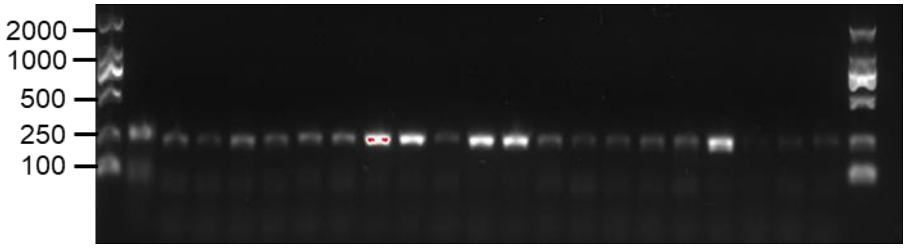

Supplement: Supplementary file 1 [file ijms-24-13825-s001.zip › Supplementary Figures.pdf]
